# 1-Cyano-*N*-(2,4,5-trichloro­phen­yl)cyclo­propane-1-carboxamide

**DOI:** 10.1107/S1600536811026225

**Published:** 2011-07-06

**Authors:** Hui-Jun Liu, Jian-Quan Weng, Cheng-Xia Tan, Xing-Hai Liu

**Affiliations:** a College of Chemical Engineering and Materials Science, Zhejiang University of Technology, Hangzhou 310014, People’s Republic of China

## Abstract

In the title compound, C_11_H_7_Cl_3_N_3_O, the dihedral angle between the benzene and cyclo­propane rings is 85.8 (2)°. In the crystal, mol­ecules are linked by C—H⋯O inter­actions, generating *C*(5) chains propagating in the *a*-axis direction.

## Related literature

For the synthesis, see: Liu *et al.* (2007 ▶). For the biological activity of related compounds, see: Liu *et al.* (2009 ▶).
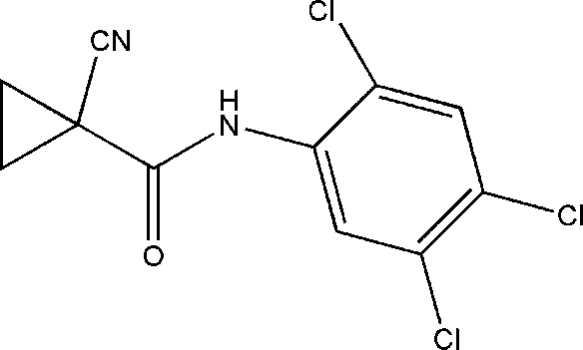


## Experimental

### 

#### Crystal data

C_11_H_7_Cl_3_N_2_O
*M* *_r_* = 289.54
Triclinic, 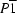

*a* = 6.0068 (18) Å
*b* = 7.420 (2) Å
*c* = 14.047 (4) Å
α = 77.531 (5)°
β = 86.958 (5)°
γ = 84.483 (5)°
*V* = 608.1 (3) Å^3^
*Z* = 2
Mo *K*α radiation
μ = 0.74 mm^−1^
*T* = 294 K
0.24 × 0.22 × 0.18 mm

#### Data collection

Rigaku Mercury CCD diffractometer
Absorption correction: multi-scan (*CrystalClear*; Rigaku/MSC, 2005 ▶) *T*_min_ = 0.614, *T*_max_ = 1.000
3103 measured reflections
2130 independent reflections
1619 reflections with *I* > 2σ(*I*)
*R*_int_ = 0.025

#### Refinement

*R*[*F* ^2^ > 2σ(*F* ^2^)] = 0.035
*wR*(*F* ^2^) = 0.107
*S* = 1.04
2130 reflections
154 parameters
H-atom parameters constrained
Δρ_max_ = 0.23 e Å^−3^
Δρ_min_ = −0.22 e Å^−3^

### 

Data collection: *CrystalClear* (Rigaku/MSC, 2005 ▶); cell refinement: *CrystalClear*; data reduction: *CrystalClear*; program(s) used to solve structure: *SHELXS97* (Sheldrick, 2008 ▶); program(s) used to refine structure: *SHELXL97* (Sheldrick, 2008 ▶); molecular graphics: *SHELXTL* (Sheldrick, 2008 ▶); software used to prepare material for publication: *SHELXTL*.

## Supplementary Material

Crystal structure: contains datablock(s) global, I. DOI: 10.1107/S1600536811026225/hb5931sup1.cif

Structure factors: contains datablock(s) I. DOI: 10.1107/S1600536811026225/hb5931Isup2.hkl

Supplementary material file. DOI: 10.1107/S1600536811026225/hb5931Isup3.cml

Additional supplementary materials:  crystallographic information; 3D view; checkCIF report

## Figures and Tables

**Table 1:** Hydrogen-bond geometry (Å, °)

*D*—H⋯*A*	*D*—H	H⋯*A*	*D*⋯*A*	*D*—H⋯*A*
C10—H10*B*⋯O1^i^	0.97	2.56	3.439 (3)	151

Symmetry code: (i) 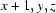.

**Fig. 1.**
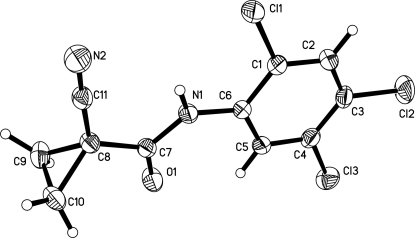
The molecular structure of (I). Displacement ellipsoids are drawn at the 30% probability level and H atoms are shown as small spheres of arbitrary radius.

**Fig. 2.**
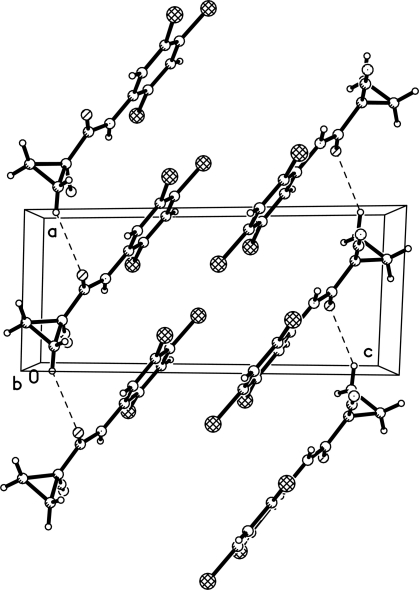
The crystal packing for (I).

## supplementary crystallographic information

### Comment

Many cyclopropane compound exhibit good biological activity such as KARI (Liu *et al.*, 2007; Liu *et al.*, 2009). In continuation of this work, the title compound, (I), a 1-cyano-carboxamide derivatives had been synthesized. The strucuture was confirmed by X-ray crstallography.

Single-crystal X-ray diffraction analysis reveals that the title compound crystallizes in the triclinic space group P1 (Fig. 1). As shown in Fig. 2, the crystal structure is stabilized by weak C-H···O intermolecular interactions.

### Experimental

The title compound was prepared according to the literature procedures (Liu *et al.*, 2007).  Colourless prisms of (I) were grown from slow evaporation of ethanol solution at room temperature.

### Refinement

All the H atoms were positioned geometrically (C—H = 0.93–0.97 Å) and refined as riding with *U*_iso_(H) = 1.2*U*_eq_(C) or 1.5*U*_eq_(methyl C).

### Crystal data

**Table:**

C_11_H_7_Cl_3_N_2_O	*Z* = 2
*M**_r_* = 289.54	*F*(000) = 292
Triclinic, *P*1	*D*_x_ = 1.581 Mg m^−^^3^
Hall symbol: -P 1	Mo *K*α radiation, λ = 0.71073 Å
*a* = 6.0068 (18) Å	Cell parameters from 1405 reflections
*b* = 7.420 (2) Å	θ = 3.0–26.2°
*c* = 14.047 (4) Å	µ = 0.74 mm^−^^1^
α = 77.531 (5)°	*T* = 294 K
β = 86.958 (5)°	Prism, colorless
γ = 84.483 (5)°	0.24 × 0.22 × 0.18 mm
*V* = 608.1 (3) Å^3^	

### Data collection

**Table:**

Rigaku Mercury CCD diffractometer	2130 independent reflections
Radiation source: fine-focus sealed tube	1619 reflections with *I* > 2σ(*I*)
graphite	*R*_int_ = 0.025
phi and ω scans	θ_max_ = 25.0°, θ_min_ = 1.5°
Absorption correction: multi-scan (*CrystalClear*; Rigaku/MSC, 2005)	*h* = −6→7
*T*_min_ = 0.614, *T*_max_ = 1.000	*k* = −8→7
3103 measured reflections	*l* = −16→16

### Refinement

**Table:**

Refinement on *F*^2^	Primary atom site location: structure-invariant direct methods
Least-squares matrix: full	Secondary atom site location: difference Fourier map
*R*[*F*^2^ > 2σ(*F*^2^)] = 0.035	Hydrogen site location: inferred from neighbouring sites
*wR*(*F*^2^) = 0.107	H-atom parameters constrained
*S* = 1.04	*w* = 1/[σ^2^(*F*_o_^2^) + (0.0581*P*)^2^ + 0.1114*P*] where *P* = (*F*_o_^2^ + 2*F*_c_^2^)/3
2130 reflections	(Δ/σ)_max_ < 0.001
154 parameters	Δρ_max_ = 0.23 e Å^−^^3^
0 restraints	Δρ_min_ = −0.22 e Å^−^^3^

### Special details

**Table:**

Geometry. All e.s.d.'s (except the e.s.d. in the dihedral angle between two l.s. planes) are estimated using the full covariance matrix. The cell e.s.d.'s are taken into account individually in the estimation of e.s.d.'s in distances, angles and torsion angles; correlations between e.s.d.'s in cell parameters are only used when they are defined by crystal symmetry. An approximate (isotropic) treatment of cell e.s.d.'s is used for estimating e.s.d.'s involving l.s. planes.
Refinement. Refinement of *F*^2^ against ALL reflections. The weighted *R*-factor *wR* and goodness of fit *S* are based on *F*^2^, conventional *R*-factors *R* are based on *F*, with *F* set to zero for negative *F*^2^. The threshold expression of *F*^2^ > σ(*F*^2^) is used only for calculating *R*-factors(gt) *etc*. and is not relevant to the choice of reflections for refinement. *R*-factors based on *F*^2^ are statistically about twice as large as those based on *F*, and *R*- factors based on ALL data will be even larger.

### Fractional atomic coordinates and isotropic or equivalent isotropic displacement parameters (Å^2^)

**Table:**

	*x*	*y*	*z*	*U*_iso_*/*U*_eq_	
Cl1	0.32398 (14)	0.92602 (9)	0.72571 (5)	0.0647 (3)	
Cl2	−0.33513 (13)	0.75928 (12)	0.52559 (6)	0.0763 (3)	
Cl3	−0.21699 (12)	0.33282 (11)	0.61532 (6)	0.0662 (3)	
O1	0.4025 (3)	0.2145 (2)	0.84621 (14)	0.0576 (5)	
N1	0.4219 (3)	0.5242 (2)	0.79194 (14)	0.0414 (5)	
H1	0.4980	0.6139	0.7971	0.050*	
N2	0.8457 (4)	0.6623 (3)	0.90380 (19)	0.0669 (7)	
C1	0.1788 (4)	0.7600 (3)	0.69152 (17)	0.0445 (6)	
C2	0.0042 (4)	0.8156 (4)	0.62960 (17)	0.0511 (6)	
H2	−0.0315	0.9410	0.6044	0.061*	
C3	−0.1181 (4)	0.6857 (4)	0.60479 (17)	0.0487 (6)	
C4	−0.0613 (4)	0.4991 (4)	0.64223 (17)	0.0456 (6)	
C5	0.1165 (4)	0.4420 (3)	0.70298 (17)	0.0416 (6)	
H5	0.1535	0.3162	0.7265	0.050*	
C6	0.2406 (4)	0.5713 (3)	0.72921 (16)	0.0384 (5)	
C7	0.4916 (4)	0.3549 (3)	0.84540 (16)	0.0389 (5)	
C8	0.6910 (4)	0.3493 (3)	0.90622 (17)	0.0399 (5)	
C9	0.7029 (4)	0.2025 (4)	1.00043 (19)	0.0541 (7)	
H9A	0.7702	0.2326	1.0557	0.065*	
H9B	0.5788	0.1257	1.0172	0.065*	
C10	0.8503 (4)	0.1735 (3)	0.9183 (2)	0.0542 (7)	
H10A	0.8171	0.0790	0.8844	0.065*	
H10B	1.0084	0.1859	0.9229	0.065*	
C11	0.7815 (4)	0.5227 (3)	0.90572 (18)	0.0448 (6)	

### Atomic displacement parameters (Å^2^)

**Table:**

	*U*^11^	*U*^22^	*U*^33^	*U*^12^	*U*^13^	*U*^23^
Cl1	0.0982 (6)	0.0340 (4)	0.0636 (4)	−0.0070 (3)	−0.0273 (4)	−0.0075 (3)
Cl2	0.0624 (5)	0.0970 (6)	0.0646 (5)	0.0174 (4)	−0.0291 (4)	−0.0110 (4)
Cl3	0.0541 (4)	0.0776 (5)	0.0773 (5)	−0.0072 (3)	−0.0193 (3)	−0.0347 (4)
O1	0.0623 (12)	0.0366 (10)	0.0747 (12)	−0.0121 (8)	−0.0290 (9)	−0.0040 (9)
N1	0.0473 (11)	0.0293 (10)	0.0494 (11)	−0.0057 (8)	−0.0149 (9)	−0.0083 (9)
N2	0.0657 (15)	0.0534 (15)	0.0857 (18)	−0.0150 (12)	−0.0214 (13)	−0.0152 (13)
C1	0.0563 (15)	0.0387 (13)	0.0391 (13)	−0.0025 (11)	−0.0064 (11)	−0.0093 (10)
C2	0.0622 (17)	0.0453 (15)	0.0418 (14)	0.0097 (12)	−0.0073 (12)	−0.0054 (12)
C3	0.0449 (14)	0.0644 (17)	0.0354 (13)	0.0079 (12)	−0.0098 (10)	−0.0111 (12)
C4	0.0426 (14)	0.0563 (15)	0.0419 (13)	−0.0019 (11)	−0.0058 (11)	−0.0193 (12)
C5	0.0447 (14)	0.0379 (13)	0.0445 (13)	0.0014 (10)	−0.0099 (11)	−0.0137 (11)
C6	0.0420 (13)	0.0376 (13)	0.0364 (12)	0.0018 (10)	−0.0060 (10)	−0.0107 (10)
C7	0.0384 (13)	0.0365 (13)	0.0429 (13)	−0.0024 (10)	−0.0065 (10)	−0.0095 (10)
C8	0.0366 (13)	0.0361 (12)	0.0470 (14)	−0.0044 (10)	−0.0061 (10)	−0.0070 (10)
C9	0.0567 (16)	0.0532 (16)	0.0497 (15)	−0.0139 (12)	−0.0155 (13)	0.0026 (12)
C10	0.0459 (15)	0.0420 (14)	0.0719 (18)	0.0037 (11)	−0.0141 (13)	−0.0063 (13)
C11	0.0405 (13)	0.0430 (14)	0.0517 (14)	−0.0039 (11)	−0.0124 (11)	−0.0090 (11)

### Geometric parameters (Å, °)

**Table:**

Cl1—C1	1.736 (2)	C4—C5	1.379 (3)
Cl2—C3	1.729 (2)	C5—C6	1.390 (3)
Cl3—C4	1.732 (2)	C5—H5	0.9300
O1—C7	1.213 (3)	C7—C8	1.500 (3)
N1—C7	1.357 (3)	C8—C11	1.442 (3)
N1—C6	1.407 (3)	C8—C9	1.522 (3)
N1—H1	0.8600	C8—C10	1.525 (3)
N2—C11	1.134 (3)	C9—C10	1.456 (4)
C1—C2	1.373 (3)	C9—H9A	0.9700
C1—C6	1.407 (3)	C9—H9B	0.9700
C2—C3	1.378 (4)	C10—H10A	0.9700
C2—H2	0.9300	C10—H10B	0.9700
C3—C4	1.386 (4)		
			
C7—N1—C6	128.11 (18)	O1—C7—C8	120.4 (2)
C7—N1—H1	115.9	N1—C7—C8	115.52 (18)
C6—N1—H1	115.9	C11—C8—C7	117.5 (2)
C2—C1—C6	121.4 (2)	C11—C8—C9	117.5 (2)
C2—C1—Cl1	119.39 (19)	C7—C8—C9	116.26 (19)
C6—C1—Cl1	119.17 (18)	C11—C8—C10	118.7 (2)
C1—C2—C3	120.1 (2)	C7—C8—C10	116.0 (2)
C1—C2—H2	120.0	C9—C8—C10	57.10 (17)
C3—C2—H2	120.0	C10—C9—C8	61.57 (16)
C2—C3—C4	119.2 (2)	C10—C9—H9A	117.6
C2—C3—Cl2	119.1 (2)	C8—C9—H9A	117.6
C4—C3—Cl2	121.6 (2)	C10—C9—H9B	117.6
C5—C4—C3	121.1 (2)	C8—C9—H9B	117.6
C5—C4—Cl3	118.6 (2)	H9A—C9—H9B	114.7
C3—C4—Cl3	120.33 (19)	C9—C10—C8	61.33 (17)
C4—C5—C6	120.4 (2)	C9—C10—H10A	117.6
C4—C5—H5	119.8	C8—C10—H10A	117.6
C6—C5—H5	119.8	C9—C10—H10B	117.6
C5—C6—C1	117.8 (2)	C8—C10—H10B	117.6
C5—C6—N1	123.8 (2)	H10A—C10—H10B	114.7
C1—C6—N1	118.4 (2)	N2—C11—C8	177.5 (3)
O1—C7—N1	124.1 (2)		
			
C6—C1—C2—C3	1.5 (4)	C7—N1—C6—C1	171.1 (2)
Cl1—C1—C2—C3	−177.48 (19)	C6—N1—C7—O1	0.1 (4)
C1—C2—C3—C4	−0.7 (4)	C6—N1—C7—C8	−179.6 (2)
C1—C2—C3—Cl2	−179.28 (19)	O1—C7—C8—C11	−176.9 (2)
C2—C3—C4—C5	−0.6 (4)	N1—C7—C8—C11	2.9 (3)
Cl2—C3—C4—C5	178.01 (18)	O1—C7—C8—C9	−30.1 (3)
C2—C3—C4—Cl3	178.07 (18)	N1—C7—C8—C9	149.7 (2)
Cl2—C3—C4—Cl3	−3.4 (3)	O1—C7—C8—C10	34.3 (3)
C3—C4—C5—C6	1.0 (4)	N1—C7—C8—C10	−146.0 (2)
Cl3—C4—C5—C6	−177.66 (18)	C11—C8—C9—C10	−107.9 (2)
C4—C5—C6—C1	−0.2 (3)	C7—C8—C9—C10	105.3 (2)
C4—C5—C6—N1	179.0 (2)	C11—C8—C10—C9	105.8 (2)
C2—C1—C6—C5	−1.0 (3)	C7—C8—C10—C9	−105.7 (2)
Cl1—C1—C6—C5	177.93 (17)	C7—C8—C11—N2	14 (7)
C2—C1—C6—N1	179.7 (2)	C9—C8—C11—N2	−132 (7)
Cl1—C1—C6—N1	−1.3 (3)	C10—C8—C11—N2	162 (6)
C7—N1—C6—C5	−8.1 (4)		

### Hydrogen-bond geometry (Å, °)

**Table:**

*D*—H···*A*	*D*—H	H···*A*	*D*···*A*	*D*—H···*A*
C10—H10B···O1^i^	0.97	2.56	3.439 (3)	151

Symmetry codes: (i) *x*+1, *y*, *z*.
